# Electrochemical Impedance Spectroscopy as a Diagnostic Tool in Polymer Electrolyte Membrane Electrolysis

**DOI:** 10.3390/ma11081368

**Published:** 2018-08-07

**Authors:** Stefania Siracusano, Stefano Trocino, Nicola Briguglio, Vincenzo Baglio, Antonino S. Aricò

**Affiliations:** CNR-ITAE, Via Salita S. Lucia sopra Contesse 5, 98126 Messina, Italy; siracusano@itae.cnr.it (S.S.); trocino@itae.cnr.it (S.T.); nicola.briguglio@itae.cnr.it (N.B.); baglio@itae.cnr.it (V.B.)

**Keywords:** water electrolysis, hydrogen generation, impedance spectroscopy, polymer electrolyte membrane, membrane-electrode assembly, oxygen evolution, iridium catalysts, platinum catalysts

## Abstract

Membrane–electrode assemblies (MEAs) designed for a polymer electrolyte membrane (PEM) water electrolyser based on a short-side chain (SSC) perfluorosulfonic acid (PFSA) membrane, Aquivion^®^, and an advanced Ir-Ru oxide anode electro-catalyst, with various cathode and anode noble metal loadings, were investigated. Electrochemical impedance spectroscopy (EIS), in combination with performance and durability tests, provided useful information to identify rate-determining steps and to quantify the impact of the different phenomena on the electrolysis efficiency and stability characteristics as a function of the MEA properties. This technique appears to be a useful diagnostic tool to individuate different phenomena and to quantify their effect on the performance and degradation of PEM electrolysis cells.

## 1. Introduction

Water electrolysis appears to be as one the foremost technologies for a sustainable production of hydrogen from renewable energy to feed next generation fuel cell vehicles. This technology is characterized by the ability to rapidly follow the intermittent production of renewable energy and, thus, can provide efficient energy storage and grid-balancing service in power-to-gas processes [1,2,3,4,5,6,7,8]. These characteristics will become very important in the next future because of the increasing impact of renewable sources on the grid and the differences in supply and demand for energy generation and consumption [1,2,3,4].

Accordingly, it is important to get a better understanding of the reaction process and performance limiting phenomena in membrane-electrode assemblies which are the core part of a PEM electrolysis system [8,9,10,11,12,13,14,15,16]. In this regard, electrochemical impedance spectroscopy appears an excellent diagnostic tool for electrochemical processes and devices especially when there is no possibility of using an internal reference electrode to help in deconvoluting different phenomena [17,18,19,20]. Ac-impedance analysis is frequently used to separate the contribution of the different mechanisms influencing the polarisation characteristics [8,20]. Electrochemical impedance spectroscopy (EIS) allows to discern between the various phenomena on the basis of the different relaxation times of the single processes and the variation of relevant components of the equivalent circuits upon a change in the operating parameters such as potential, current density, temperature and MEA characteristics, e.g., catalyst loading [17,20]. In most cases, a first qualitative analysis of electrochemical impedance spectra allows to clearly distinguish between ohmic and polarization resistance providing a first insight if the system needs to be improved in terms of electrolyte or electrodes, if there are relevant interface issues or there is an occurrence of diffusion-related phenomena [17]. However, the relevant information provided by the ac-impedance spectra would require an in-depth investigation using equivalent circuit analysis [20]. This can be carried out in combination with a systematic variation of the main variables influencing the electrochemical process such as potential, temperature, current density and the specific MEA characteristics having a major or specific impact on a single process [20].

Several groups have already used electrochemical impedance spectroscopy to interpret more in detail the observed electrochemical phenomena and to derive information useful in improving performance and durability [8,13,14,15,16,20,21,22,23,24,25,26,27,28,29,30]. However, the response of a PEM electrolysis system varies significantly with regard to the type and thickness of membrane used, the different anode electrocatalyst, the type of current collector and diffusion media etc. [4,5,6,7,8,9]. Thus, the results are often specific of the system under consideration and some phenomena are linked to the MEA configuration and materials used.

Recently, we have shown that high electrolysis current density (3 A cm^−2^) corresponding to reduced capital costs can be achieved with a low or moderate platinum group metals (PGM) catalyst loading (0.5–1.5 mg cm^−2^_MEA_) while maintaining a very high conversion efficiency (>80%) and good stability [8,25,28]. This is obtained in the presence of a nanostructured Ir-Ru electro-catalyst for oxygen evolution (the rate determining step in the electrolysis process) in combination with a short-side chain perfluorosulfonic acid membrane [8,25,28]. The stability of this system is essentially governed by the reaction turn-over frequency, i.e., the combination of the effects of the operating current density and catalyst loading [8].

To further advance in this field, it is important to get proper insights about the rate determining steps and the contribution of each process and component of the membrane-electrode assembly to the overall polarisation behaviour and the related efficiency characteristics. Thus, it is necessary to extract all relevant information from the available electrochemical characterisation to help in understanding relevant aspects such as the effect of the catalyst loading, membrane and electro-catalysts contributions to the cell polarisation, possible occurrence of mass transport limitations and degradation issues.

It is considered that, beside the conventional steady-state characterization, time-resolved techniques, such as ac-impedance spectroscopy, can provide further insights on the relevant phenomena and help to differentiate between the characteristics of the different PEM electrolysis systems to identify properly limiting steps thus allowing to develop effective solutions. The aim of this work is to get a deeper understanding of the behaviour of these PEM electrolysis MEAs by carrying out an electrochemical impedance spectroscopy study in combination with equivalent circuit analysis.

Electrochemical Impedance Spectroscopy is a powerful diagnostic tool for several electrochemical processes. However, in this method, data validation is very important to avoid interferences by non-steady state or non-linear behaviour [17,18]. In particular, an analysis of the residuals is always necessary to understand the appropriateness of the model and the resulting equivalent circuit should lead to a realistic physical interpretation [17,18]. Equivalent circuits are in general useful in analysing impedance spectra arising from reaction mechanisms and their use appears very appropriate in this context [20]. They provide a useful model to validate the data whereas the circuit elements can be used to screen the relevant mechanisms.

In this work, we have addressed all these aspects and specific attention was devoted to minimise the deviation between the experimental results and the fitting; this is shown in both Nyquist and Bode plots. We have used a very small perturbation signal to avoid non-linearities under all conditions while trying to avoid relevant data scattering [17,18]. The approach used in this work for the ac-impedance analysis was to carry sequential measurements of impedance by Fourier analysis. The electrolysis cell under investigation can be considered like a stationary system being the cell voltage very stable during galvanostatic operation. The sequence of frequencies (f) and the frequency intervals, Δf/f, were properly selected to minimise the time associated to each measurement while providing a highly resolved profile.

In this method, the measurements at each frequency are independent of each other. Their consistency with the Kramers–Kronig relations [17,18] is an important step of the validation procedure. Moreover, to corroborate the interpretation of the results and the corresponding equivalent circuits, specific parameters such as catalyst loading or temperature have been systematically varied to verify if their effect on the impedance response was in agreement with the suggested model.

## 2. Materials and Methods

### 2.1. Materials and Cells

Unsupported IrRuOx catalyst (70% at. Ir: 30% at. Ru), prepared by using a modified Adams process, was used as anode electrocatalyst [31]. A 40% Pt/Vulcan XC-72 was synthesized according to the sulphite complex process [31,32] and utilised at the cathode.

A chemically stabilised Aquivion^®^ short-side chain extruded film (E98–09S), with a thickness of 90 µm and an equivalent weight (EW) of 980 g/eq developed by Solvay Specialty Polymers, specifically for water electrolysis applications, was used as membrane separator. An ionomer dispersion (830 g/eq equivalent weight-based Aquivion^®^ ionomer—D83–06A) was used as ionic conductor in both the composite catalytic inks [28,32].

Membrane-electrode assemblies (MEAs) were manufactured by using a catalyst-coated membrane (CCM) preparation procedure [8]. Separate catalyst slurries of IrRuOx and Pt/C with the Aquivion^®^ ionomer dispersion (D83–06A) were sprayed in sequence onto the different faces of the Aquivion^®^ membrane. The ionomer content in the catalytic layers was 15 wt. % at the anode and 28 wt. % at the cathode. The properties of the catalytic inks were examined by using a combination of transmission electron microscopy and X-ray diffraction (XRD). Structural analysis was carried out by a Panalytical X-Pert diffractometer using a CuKa as radiation source operating at 40 kV and 20 mA. The diffraction patterns were interpreted using the Joint Committee on Powder Diffraction Standards (JCPDS). The morphology of the catalytic layers was investigated by transmission electron microscopy (TEM) using a FEI CM12 instrument.

CCMs were hot-pressed at 190 °C to favour hot bonding of the catalytic layers to the membrane at a temperature higher than the glass transition temperature of the Aquivion^®^ polymer. The MEA was installed in an electrolysis cell housing consisting of Ti plates. A carbon based gas diffusion layer (GDL ELAT) was used as backing layer at the cathode whereas a titanium fibre mesh (Bekaert Toko Metal Fiber Co., NV Bekaert SA Bluepoint, Brussels, Belgium) was used as diffusion layer for the anode. The PGM loading was varied as reported in the results section. The total geometrical area of each electrode (anode and cathode) was 5 cm^2^. This was assumed as the active electrode area; this value (5 cm^2^) was used to normalise the current to the specific electrode area (current density).

### 2.2. Electrochemical Studies

The performance of the PEM electrolysis cells was studied at different temperatures and at ambient pressure. Milli-Q Integral, Millipore deionized water, with a resistivity of 18 MΩ∙cm, was supplied to the anode with a flow rate of 1 mL·min^−1^ cm^−2^ and recirculated at the same temperature of the cell. The electrochemical investigation included galvanostatic polarization curves, electrochemical impedance spectroscopy (EIS), and chronopotentiometric studies under galvanostatic conditions. These studies were carried out using an electrochemical set-up made of an Autolab PGSTAT 302 Potentiostat/Galvanostat, a 20 A booster (Metrohm, Herisau, Switzerland) and FRA (frequency response analyser).

The working and sense wires of the Autolab were both connected to the anode (positive electrode) current collector whereas the counter and reference wires were connected to the cathode (negative electrode) current collector. Thus, the cell potential (sense vs. reference) was sampled directly on the cell terminals/current collectors while current was passing through the working and counter wires of the Autolab machine.

Polarization curves (cell potential versus cell current) were carried out in the galvanostatic mode by recording the cell voltage vs. the imposed current density. A stepwise logarithm increase of current was used for the galvanostatic polarization curves using a cut-off voltage of 2 V. Variation of the current density was carried out in steps and the duration of each step was 1 min (pseudo steady state-condition). The change in the cell voltage for 1 min, generated by variation of current was registered. The average potential was reported at each current density.

Electrochemical impedance spectroscopy analysis was performed at 1.5 V and 1.8 V under different conditions by varying the frequency from 100 kHz to 100 mHz in single sine mode and using a sinusoidal excitation signal of 10 mV pk-pk.

## 3. Results and Discussion

### 3.1. Structure and Morphology of the Electro-Catalytic Inks

**Figure 1:**
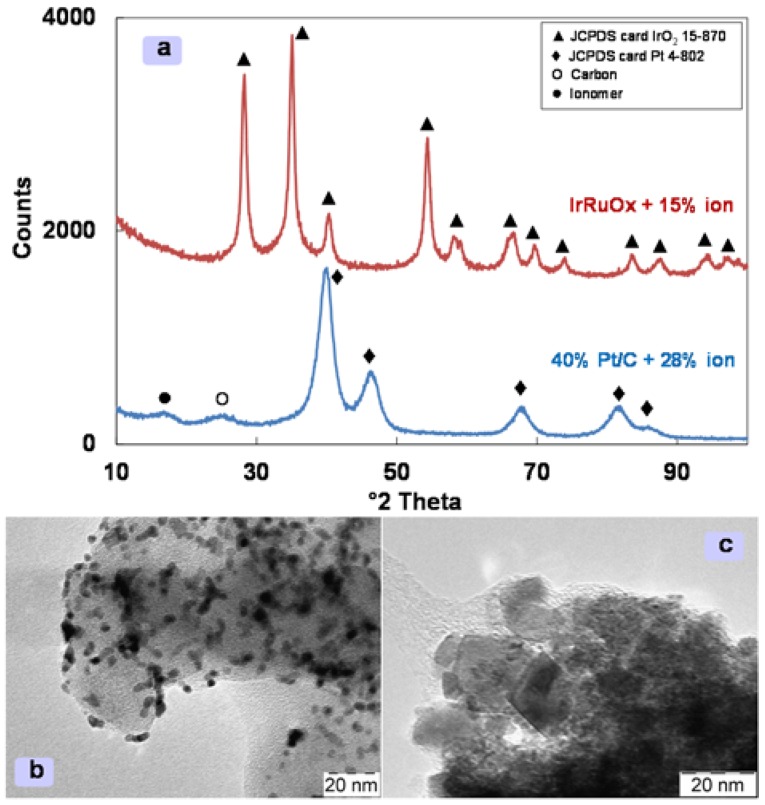
X-ray diffraction of IrRuOx +15% ionomer and 40% Pt/C + 28% ion (**a**); TEM image of 40% Pt/C + 28% ion (**b**); TEM image of IrRuOx +15% ion (**c**).

The XRD analysis of the catalyst inks shows for the mixed IrRuOx-ionomer ink the tetragonal rutile crystallographic structure of IrO_2_ with RuO_2_ forming a solid solution as suggested by the shift of the diffraction peaks towards higher Bragg angles [31]. There is a minimal evidence of the ionomer peak at 16° 2-theta (Figure 1a). From the broadening of the XRD peaks a mean crystallite size of 8 nm is determined. The amount of Aquivion^®^ ionomer in the anode ink is minimized to avoid hindering electronic percolation in the oxide anode [20]. On the other hand, the ionomer peak is well evident in the catalytic ink of the cathode due to the larger ionomer content used in the presence of the carbon supported Pt catalyst (Figure 1). This shows the conventional face-cantered cubic structure of Pt with a crystallite size of 3 nm as determined from the X-ray peaks broadening.

Ionomer dispersion around the catalyst agglomerates is observed in the TEM pictures of the catalytic inks (Figure 1b,c). The ionomer forms a film around the catalyst agglomerates without affecting significantly electronic percolation while providing network for ionic conduction inside the catalytic ink. This is a prerequisite to obtain a proper extension of the catalyst-electrolyte interface inside the catalytic layer. Gas evolution thus occurs at the triple-phase boundary between catalyst-electrolyte and gas phase. The Pt particles appear uniform in size whereas the IrRuOx is composed of both fine and large particles. The latter however are not larger than 20 nm.

### 3.2. Operating Regime of a PEM Electrolyser

Polarisation studies are carried out at 80 °C in a current density range where the voltage efficiency is appropriate for practical applications (>70% vs. H_2_ high heating value). Depending on the operating temperature this range varies up to 2 or 3 A cm^−2^. Ac-impedance spectra are collected at two operating cell voltages, 1.5 V and 1.8 V, which correspond to the system operation under activation or ohmic-diffusion control, respectively. The first part of this study deals with cell operation at 1.5 V to get a more in depth understanding of the catalytic processes. The second part is related to the operation at high potentials where ohmic and diffusion constraints affect the normal operation of the PEM electrolysis system at the nominal current density.

Generally, the nominal operating current density of a PEM electrolysis system is deriving from a compromise between hydrogen production rate, durability and efficiency [8,32]. The hydrogen production is linked to the operating current density according to the Faraday’s law. Operation at higher currents reduces capital costs because the same stack hardware is used to produce more hydrogen per unit of time [8,32]. However, operation at very high current density reduces the voltage efficiency and increases the degradation rate [8]. These characteristics influence the operating expenditures; operation at higher reaction rate but lower efficiency can be less useful when the cost of electricity is high. All these aspects influence the final cost of the produced hydrogen.

A practical system operates at conditions where capital costs and operating expenditures are acceptable in the specific context where the electrolyser is installed [32]. The ac-impedance analysis at low current density is of practical interest because of the possibility of partial load operation in grid-balancing service and the fact that the observed phenomena mainly reflect the electrocatalyst behaviour. These are of relevant interest since there is always the need to minimize the PGM loading without affecting considerably performance and stability.

Instead ac-impedance analysis at high current densities is very useful to get information on the limiting steps during operation at nominal current density. Besides, the operating temperature of a PEM electrolyser can vary depending on the stack and system design as well as a function of the active materials involved. Accordingly, an impedance study should cover these variables. Moreover, the operating pressure also plays an important role since a practical electrolyser usually operates in a pressurized mode [1]; however, this effect is not studied in this work.

### 3.3. Effect of Different Cathode Loadings

**Figure 2:**
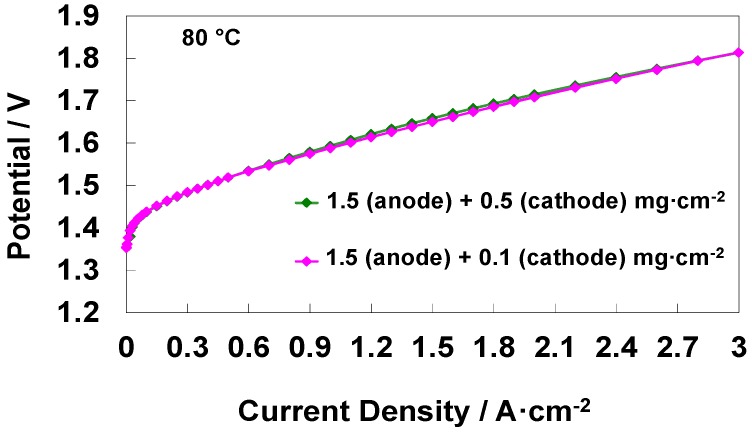
Comparison of the water electrolysis polarization curves for different cathode catalyst loading-based MEAs at 80 °C.

**Figure 3:**
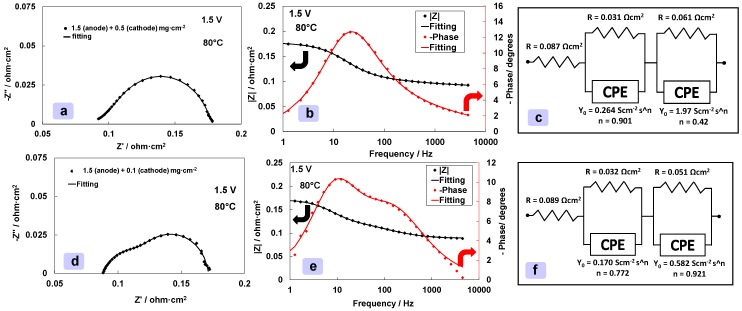
EIS at 1.5 V and 80 °C for high (**a**–**c**), 0.5 mg cm^−2^, and medium (**d**–**f**), 0.1 mg cm^−2^, cathode catalyst loading MEAs. Nyquist plots are reported in (**a**) and (**d**). Bode plots are reported in (**b**) and (**e**), equivalent circuits are reported in (**c**) and (**f**).

Polarisation curves and ac-impedance were both used to assess the electrochemical behaviour of electrolysis MEAs (Figure 2 and Figure 3). For what concerns the polarization curves (Figure 2), an increase of cell potential at a fixed current density means a decrease of voltage efficiency since more energy is used to produce the same amount of hydrogen.

A first qualitative analysis of Nyquist spectra (Figure 3a,d) allows to distinguish between series or ohmic resistance and polarization resistance. The high frequency intercept of the semicircles on the *x*-axis in the Nyquist plots is associated to the ohmic resistance (more precisely reported as series resistance, R_s_). The difference between the low frequency intercept in the Nyquist plot and R_s_ is assumed as the polarisation resistance (R_p_). The total resistance corresponding to the low frequency intercept on the abscissa in the Nyquist plot (R_s_ + R_p_) is corresponding to the differential resistance of the polarization curves.

Polarisation curves of MEAs containing constant anode loading but cathode catalyst loading varying five-times (0.1 and 0.5 mg cm^−2^) are shown in Figure 2. It is interesting to note that the curves are overlapping at all current densities indicating no influence of the Pt C loading. However, the shape of the Nyquist and Bode plots obtained from the electrochemical ac-impedance analysis of the two cells at 1.5 V cell voltage, i.e., in a region where the catalytic effects should be relevant, are significantly different (Figure 3a–e). Figure 3 shows both the experimental ac-impedance data and related curve fitting in Nyquist (a,d) and Bode mode (b,e). Figure 3c,f show the equivalent electrical circuits used to fit the ac-impedance data obtained for the high and low-cathode loaded MEA. The equivalent electrical circuits include an ohmic resistance (series resistance) connected in series with two components each consisting of a parallel between a resistance and a constant phase element (CPE) [18,26]. The series resistance reflects the ohmic phenomena whereas the RQ components are associated to the electrode-electrolyte interfacial properties and related faradaic processes [18,26]. In particular, the constant phase element, here represented with the Q symbol, reflects the fractal nature and the roughness of the interface between the electrocatalyst and the ionomer electrolyte [17,18,19,20].

In both cases, there are two overlapping semicircles in the Nyquist plots (Figure 3a,d) corresponding to two different relaxation times in the Bode plots in Figure 3b,e. These are represented by two overlapping peaks in the curves related to the phase shift. A small semicircle is occurring at high frequencies in both cases. This is characterized by small resistance especially when the Pt loading in the cathode is relatively large. The resistance associated to this semicircle increases considerably when the Pt loading at the cathode is reduced from 0.5 to 0.1 mg cm^−2^. This evidence clearly indicates that the cathode process is responsible for the high frequency arc. The arc occurring at low frequency and characterized by higher polarization resistance, even when the cathode catalyst loading is substantially reduced, is attributed to the anode (the oxygen evolution is the slowest mechanism in the electrolysis process). The presence of a high Pt content at the cathode thus reduces significantly the high frequency semicircle in the Nyquist plot (Figure 3a) and its related relaxation time peak (Figure 3b) is not well evident in the Bode plot. In the literature, the high frequency arc in the Nyquist plot of a PEM electrolysis MEA is attributed either to the hydrogen evolution process or to a charge transfer process combining double layer effects of the ionomer and oxides in the active anode layer [33]. However, because of the very low cathode catalyst loading, in the present case, the hydrogen evolution process seems to have a relevant effect on the high frequency semicircle.

Interestingly, the main variation that is observed in the Nyquist plot is essentially related to the capacitive effects at high frequency but there is essentially no change in the overall resistance (low frequency intercept on the *x*-axis). Thus, the polarization curves are overlapping in this region. It is evident that the anode contribution is dominating, in both cases, the overall polarization behaviour. The anode contribution is almost comparable to the contribution of the series resistance in this range of current densities (Figure 3c,f). In general, ac-impedance spectra reveal features that the polarization curves do not make evident.

### 3.4. Electrochemical Characterization of an Electrolysis MEA at Different Temperatures

**Figure 4:**
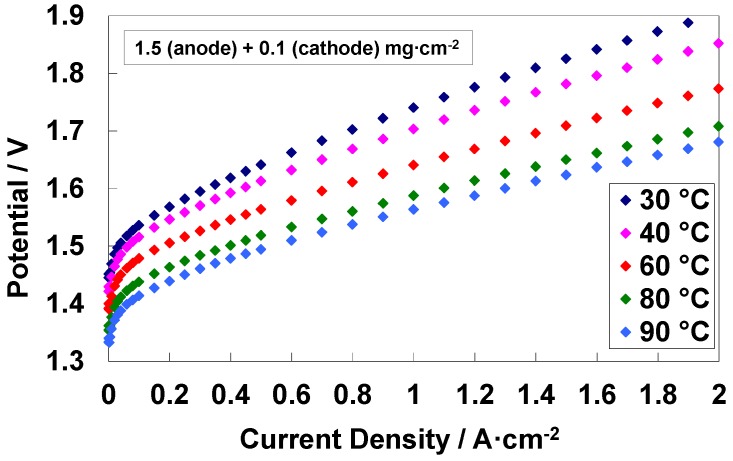
Polarization curves at different temperatures for the medium catalyst loading (1.6 mg cm^−2^_MEA_)-based MEA.

Focusing on the MEA having a low Pt content, a set of polarization curves at increasing temperatures is shown in Figure 4. Two relevant features can be observed: (i) the increase of cell potential at low current density (activation control) is more relevant as the temperature decreases, (ii) the slope of the polarization curves increase as the temperature decreases. Both effects are related to a variation of efficiency. The first aspect is essentially related to the activation process and it is associated to the catalytic system and the reactions involved; the second aspect is related to the proton conductivity of the membrane that it is also a temperature-activated process [34,35].

**Figure 5:**
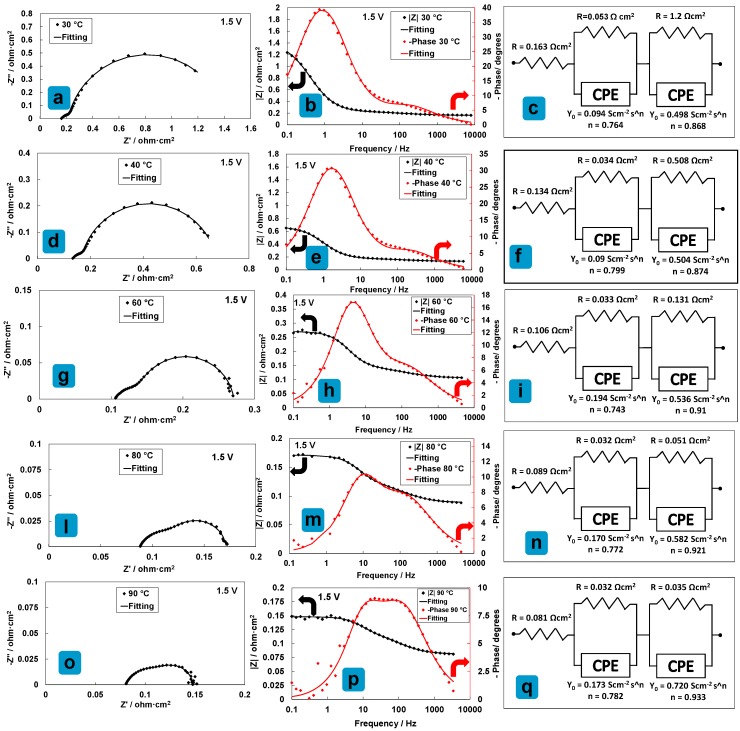
EIS analysis at 1.5 V and increasing temperatures for the medium catalyst loading (1.6 mg cm^−2^_MEA_)-based MEA. Nyquist plots are reported in (**a**,**d**,**g**,**l**,**o**); Bode plots are reported in (**b**,**e**,**h**,**m**,**p**); equivalent circuits are reported in (**c**,**f**,**i**,**n**,**q**).

**Figure 6:**
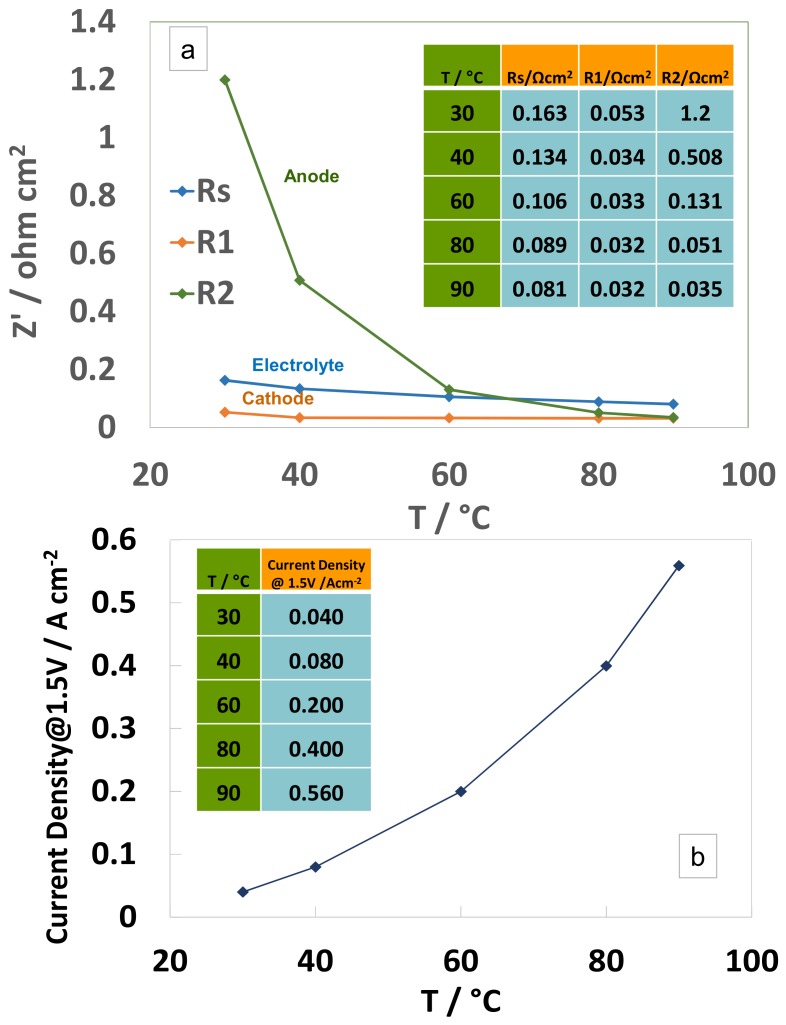
Variation of relevant electrochemical parameters at 1.5 V as a function of temperature for the medium catalyst loading (1.6 mg cm^−2^_MEA_)-based MEA. (**a**) R_s_ refers to the series resistance; R1 and R2 refer to the high and low frequency relaxation processes, respectively. (**b**) Variation of current density with temperature at 1.5 V.

To understand the effect of temperature on the catalytic processes at low current density, ac-impedance spectra collected at 1.5 V and different temperatures are shown in Figure 5 with reference to the low cathode loading MEA. For this MEA with low Pt loading, the Nyquist plots clearly show that the high frequency arc is not much affected by the temperature differently from the series resistance and the low frequency arc. Quantification of the resistance associated to the high frequency arc according to the curve fitting procedure and related equivalent circuit analysis indicates a moderate decrease from 0.053 Ohm cm^2^ at 30 °C to 0.032 Ohm cm^2^ at 80 °C (Figure 6a). On the other hand, the resistance associated to the low frequency arc associated to the anode reaction decreases from 1.2 to 0.035 Ohm cm^2^ (almost one order of magnitude) in the same temperature range (Figure 6a). The series resistance, associated mainly to the membrane, decreases by one half, i.e., from 0.163 Ohm cm^2^ at 30 °C to 0.081 Ohm cm^2^ at 80 °C. According, to this analysis, the anode reaction benefits much more than the other two processes from the increase of temperature (Figure 6a). Figure 6b clearly shows that the increase of cell current density at 1.5 V with temperature is inversely proportional to the decrease of the anode polarization resistance. Moreover, at 90 °C, the sum of the contributions of the electrodes to the overall resistance is slightly lower than the contribution from the electrolyte (Figure 6a).

Another interesting aspect is that the relaxation frequencies for the anode and the cathode, which are well separated at 30 °C, become similar at 90 °C (Figure 5b,p). This evidence and the values of the polarization resistances recorded at 90 °C indicate that the oxygen evolution process is characterized by a reaction rate similar to that of the hydrogen evolution process at 90 °C. Accordingly, an important strategy to increase the performance of the PEM electrolyser would be to increase the operating temperature provided that the stability is not affected.

### 3.5. Analysis of an Electrolysis MEA with Reduced Anode and Cathode Catalyst Loadings

**Figure 7:**
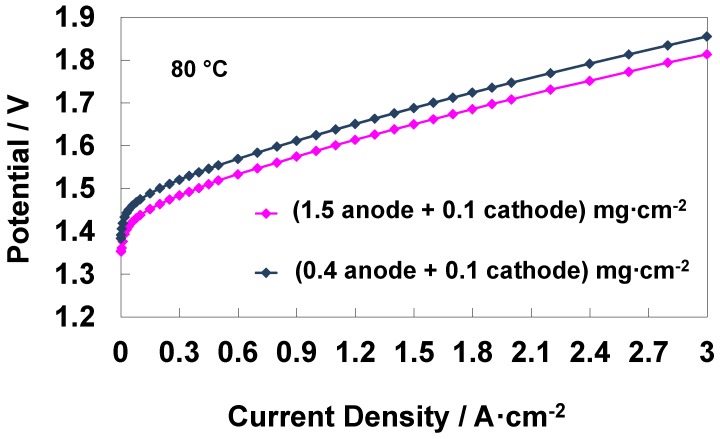
Comparison of the water electrolysis polarization curves for different anode catalyst loading -based MEAs at 80 °C.

**Figure 8:**
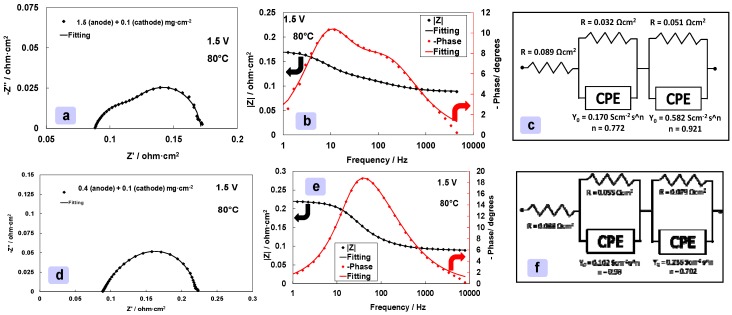
EIS at 1.5 V and 80 °C for high (**a**–**c**), 1.5 mg cm^−2^, and medium (**d**–**f**), 0.4 mg cm^−2^, anode catalyst loading MEAs.

When the anode loading is decreased by about three times, from 1.5 to 0.4 mg cm^−2^, the activation losses at low current density increase by 30–40 mV and this is reflected in the overall range of current density (Figure 7). The slopes of the polarization curves at high current density are essentially determined by the membrane but their onset potential is affected by the content of the anode electrocatalyst in the investigated range (Figure 7). Electrochemical impedance spectroscopy data (Figure 8a–f) show that a decrease of the anode loading causes an increase in the corresponding polarization resistance that overlaps to the cathode process. In comparison, the polarization resistance of the cathode almost disappears (Figure 8a,d). The same effect is observed for the relaxation frequencies in the Bode plot (Figure 8b,e). The overall resistance increases in this case (Figure 8) and this is reflected in the relevant change of the slope in the polarization curves at low current densities for the low anode loading MEA (Figure 7).

### 3.6. Effect of the Operating Potential Windows

**Figure 9:**
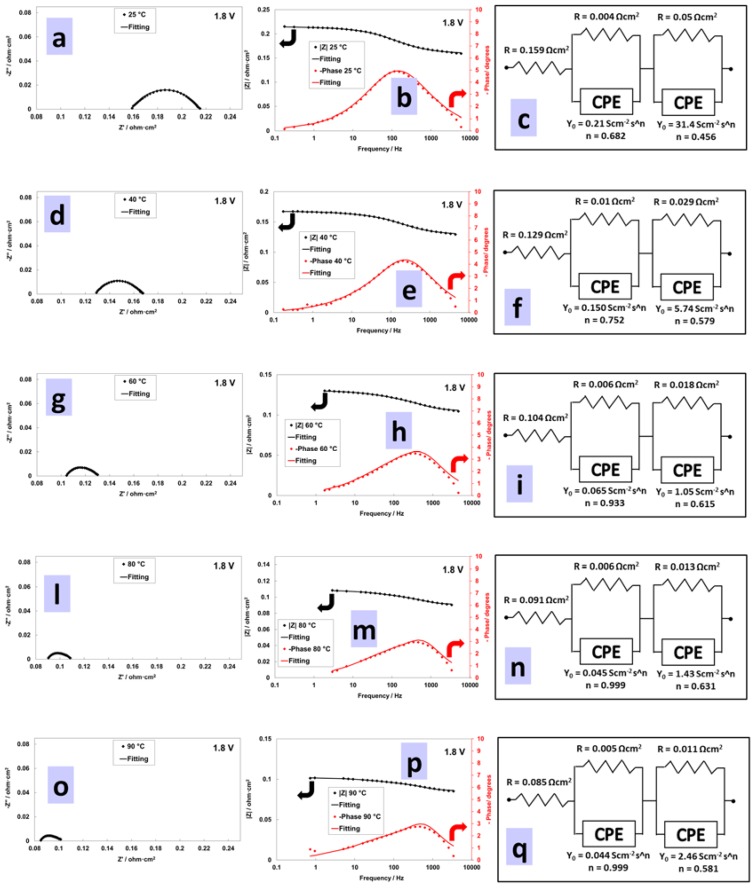
EIS at 1.8 V for low catalyst loading at 25 °C (**a**–**c**), 40 °C (**d**–**f**), 60 °C (**g**–**i**), 80 °C (**l**–**n**), 90 °C (**o**–**q**).

**Figure 10:**
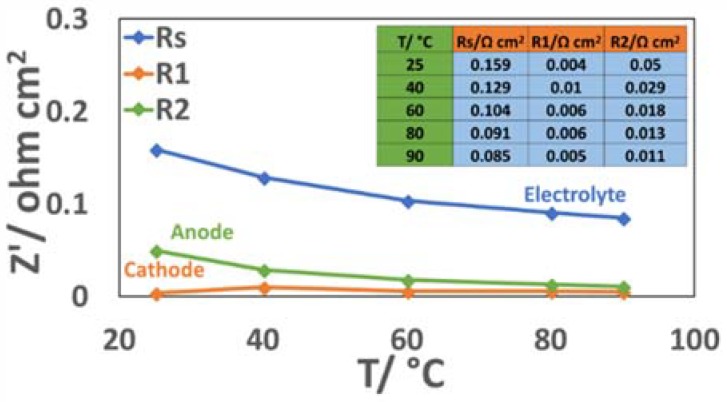
Variation of relevant electrochemical parameters at 1.8 V as a function of temperature for the low catalyst loading-based MEA. Rs refers to the series resistance; R1 and R2 refer to the high and low frequency relaxation processes, respectively.

Figure 9 shows the ac-impedance spectra collected at 1.8 V for the MEA characterized by low catalyst loadings. The series resistance associated to the membrane contribution is dominating the overall polarization behaviour at the different temperatures. The role of the membrane is significant (>80% contribution) at higher current density and high temperatures (Figure 9 and Figure 10). The overall polarization resistance (difference between high and low frequency intercept) decreases more than three times, from 0.06 to 0.015 Ohm cm^2^, passing from 25 °C to 90 °C whereas series resistance is reduced to one half from 0.16 to 0.08 Ohm cm^2^ (Figure 9 and Figure 10). Thus, the polarization resistance is more affected by the increase of temperature in terms of relative variation, whereas the membrane resistance, being dominant, is more influenced by the temperature in terms of absolute values. The curve fitting is still sufficiently valid using two semicircles in series with the ohmic (membrane) resistance. However, being the hydrogen process characterized by a very low resistance at high current density, a precise estimation of its contribution is not easy (Figure 9). The same for the relaxation times in the Bode plots (Figure 9). A relaxation frequency at high frequency is envisaged from the experimental data (Figure 9); however, the curve fitting slightly deviates from the experimental data in that range failing to determine the exact hydrogen relaxation frequency (this is shifted to very high frequencies where membrane effects are dominating the process). Finally, at 90 °C and 1.8 V (Figure 9), some deviation of the curve fitting from the experimental data at low frequency may suggest the onset of a mass transfer controlled process. Unfortunately, measurements at very low frequency are significantly affected by noise in the presence of a large gas evolution.

### 3.7. Analysis of MEA Degradation

**Figure 11:**
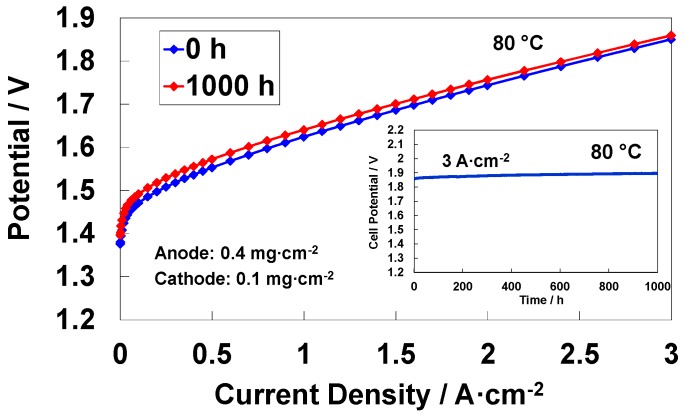
Polarization curves at 80 °C before and after a 1000 h durability test for a low-loading MEA (both low anode 0.4 mg cm^−2^ and cathode 0.1 mg cm^−2^ loadings). The inset shows the durability tests at 3 A·cm^−2^.

Electrochemical impedance spectroscopy is also a powerful tool to understand the degradation of an MEA in a durability experiment (Figure 11). A time-study at 3 A cm^−2^ was thus carried out for the MEA characterised by the low catalyst loading at both electrodes (total PGM loading 0.5 mg cm^−2^) showing a degradation rate of about 20 µV/h in a 1000-h durability test according to a best fit procedure (Figure 11 inset). The polarization curves before and after the durability test show an increase of cell potential slightly smaller than 20 mV at low current densities after the time-study. However, this loss is in part recovered at high current densities.

**Figure 12:**
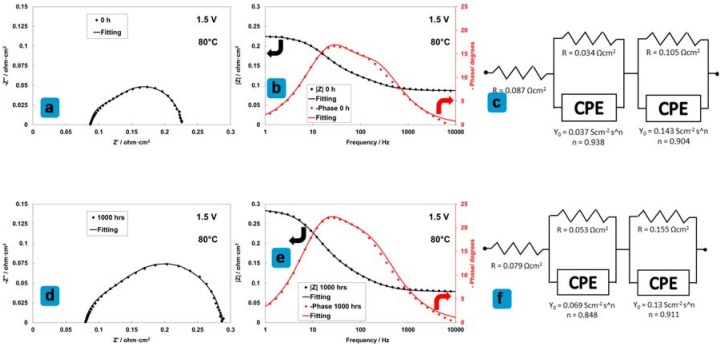
EIS at 1.5 V and 80 °C, before and after a 1000 h durability test at 3 A cm^−2^, for a low-loading MEA (both low anode 0.4 mg cm^−2^ and cathode 0.1 mg cm^−2^ loadings). Nyquist plots are reported in (**a**,**d**); Bode plots are reported in (**b**,**e**); equivalent circuits are reported in (**c**,**f**).

Figure 12 shows that after 1000 h of operation at 3 A cm^−2^, there is a substantial increase of the anode polarization resistance at low frequency (from 0.1 to 0.17 Ohm cm^2^). A moderate increase of the high frequency arc (from 0.03 to 0.04 Ohm cm^2^) is also observed whereas the series (ohmic) resistance (high frequency intercept on the abscissa in the Nyquist plot) shows a very small decrease. This decrease (from 0.09 to 0.08 Ohm cm^2^) is, however, in part sufficient to allow recovering at high current density some of the performance loss caused by the anode deactivation. As discussed above, the membrane plays a dominant role at high operating currents. This decrease of the membrane resistance may be associated with some restructuring of the membrane during operation at high currents. The impedance spectroscopy shows that there is a degradation for both anode and cathode but the first is more pronounced. This is somewhat expected because the anode operates in a much higher potential window.

## 4. Conclusions

This work shows that electrochemical impedance spectroscopy can provide relevant information to identify and quantify the contribution of the electrode processes to the polarization behaviour of a PEM electrolysis cell. This is especially useful when the single cell or a stack cannot be equipped with an internal reference electrode, either for the specific design used or for the operating conditions e.g., high pressure. Ac-impedance spectra are collected under the specific operating conditions of the electrolysis cell and, thus, this technique is not invasive. It supplies important information on the limiting phenomena and it can provide useful insights to drive the research efforts towards a specific cell component in the case of post-operation degradation studies.

The present study has shown that membrane resistance plays a dominant role at high current density, which is usually the nominal operating condition under stationary mode, whereas the anode process is relevant under partial load operation. The latter condition is frequently occurring under grid-balancing service. An increase of temperature strongly activates all these processes, especially the anode reaction. At 90 °C, the anode contribution to the polarisation resistance is not significantly higher than that associated to the cathode, whereas at low temperature and low potentials the anode process is the rate determining step.

The registration of ac-impedance spectra during the course or at the end of durability studies provides a non-invasive tool to identify which component is more affected by degradation. In the present study, the anode was identified to contribute to the degradation behaviour in a more pronounced extent than the cathode, whereas the decrease of the series resistance would suggest some restructuring of the polymer during operation but, in some cases, it may be associated to a membrane thinning effect. Thus, an analysis of the evolution of the EIS spectra during cell or stack operation may provide useful insights into the state of the electrolysis system serving as a non-invasive diagnostic tool.
